# Examination of the Five Comparable Component Scores of the Diet Quality Indexes HEI-2005 and RC-DQI Using a Nationally Representative Sample of 2–18 Year Old Children: NHANES 2003–2006

**DOI:** 10.1155/2013/376314

**Published:** 2013-09-15

**Authors:** Sibylle Kranz, George P. McCabe

**Affiliations:** ^1^Department of Nutrition Science, Purdue University, 204 Stone Hall, 700 W. State Street, West Lafayette, IN 47907, USA; ^2^Department of Statistics, College of Science, Purdue University, 932 Mathematical Sciences Building, 150 N. University Street, West Lafayette, IN 47907-2067, USA

## Abstract

Obesity has been associated with low diet quality and the suboptimal intake of food groups and nutrients. Two composite diet quality measurement tools are appropriate for Americans 2–18 years old: the Healthy Eating Index (HEI) 2005 and the Revised Children's Diet Quality Index (RC-DQI). The five components included in both indexes are fruits, vegetables, total grains, whole grains, and milk/dairy. Component scores ranged from 0 to 5 or 0 to 10 points with lower scores indicating suboptimal intake. To allow direct comparisons, one component was rescaled by dividing it by 2; then, all components ranged from 0 to 5 points. The aim of this study was to directly compare the scoring results of these five components using dietary data from a nationally representative sample of children (NHANES 2003–2006, *N* = 5,936). Correlation coefficients within and between indexes showed less internal consistency in the HEI; age- and ethnic-group stratified analyses indicated higher sensitivity of the RC-DQI. HEI scoring was likely to dichotomize the population into two groups (those with 0 and those with 5 points), while RC-DQI scores resulted in a larger distribution of scores. The scoring scheme of diet quality indexes for children results in great variation of the outcomes, and researchers must be aware of those effects.

## 1. Introduction

The development of diet quality indexes began several decades ago when capturing the characteristics of complete diets, rather than consumption levels of specific nutrients, became a goal in nutrition monitoring. This progression away from the minimalist's approach addressed the need for tools to measure differences between diets of individuals and the dietary intake recommendations or other dietary standards. Unlike in animal-based research, which can be conducted in settings that allow complete control, free living people consume foods at various locations and throughout the day—most often not following obvious patterns. Thus, total food intake is a complex construct, which cannot be described or evaluated based on nutrients measured in isolation of one another. Even within foods, nutrients have complex synergistic effects upon one another that are not always well understood [1]. Therefore, considering intake of nutrients and their relationship to overall health and disease may be misleading where indexes measuring consumption levels of food groups and nutrients, concurrently, are superior approaches to estimating overall diet quality.

Currently, a number of diet quality indexes exist. Several of them were adapted to reflect the nutritional needs of different population groups. Two of those are the Healthy Eating Index (HEI) [2] and Diet Quality Index (DQI) [3]. The HEI was first developed in 1995 to measure diet quality of Americans two years and older in terms of compliance with newly released Federal Guidelines and was used by the USDA for nutrition monitoring. It was revised (HEI-2005) to reflect changes introduced by the 2005 Dietary Guidelines for Americans (DGA) [4]. Currently, the HEI-2005 is revised again to reflect the DGA 2010 (release is expected late in 2012). 

The HEI 2005 has 12 components, representing both adequacy and moderation, with maximum scores ranging from 5 to 20 points, all adding up to a total of 100 possible points. All HEI components are assessed on a density basis, that is, percentage of calories per 1000 calories consumed, allowing for characterization of intake levels while controlling for total energy intake, which is highly correlated with the quantity of foods consumed. The use of energy-adjusted estimates was an important difference from the HEI 1995 version and addressed the premise that a person eating a lot of food would be much more likely to meet minimum food group or nutrient intake levels than a person consuming less food (and energy). Maximum points reflect meeting or exceeding the intake standards, zero points indicate that the individual did not consume the recommended level of the food group/nutrient, and scores between the two extremes are prorated in a linear fashion. The intake standards for adequate intake were based on MyPyramid recommendations. For moderation components, the standard was set at the 85th percentile of the population distribution in order to prevent a large proportion of the population from receiving a zero score [5].

Performance of the HEI-2005 was evaluated for content and construct validity and reliability by scoring the reported one-day intakes of 8,650 individuals and by scoring menus. Content validity was measured by comparing each HEI-2005 component to key diet quality recommendations in the 2005 Dietary Guidelines for Americans. Construct validity was measured by scoring exemplary menus, theoretically capturing a high quality diet, and reliability was assessed through tests of internal consistency. Results showed strong evidence that the HEI-2005 is a valid measure of diet quality and most components are independent of energy intake [6].

The original DQI was developed for Americans two years old and up in 1994 as the composite measure of diet quality with eight components, each scored from a zero to two with zero being the measure of meeting standards [3]. In 1999, the DQI was revised (DQI-R) to reflect the new dietary guidance given by the 1995 Dietary Guidelines for Americans and modeled moderation, variety, and proportionality within a 100 point framework, with a higher score being indicative of a higher diet quality [7]. Just as the HEI, the RC-DQI was developed using two-day averages of dietary intake data from the nationally representative data sets from the Continuing Survey of Food Intake by Individuals (CSFII) or the National Health and Nutrition Examination Survey (NHANES), with food group data from the MyPyramid Equivalents Database (MPED). 

Children are physiologically different from adults, and having a high quality diet is challenging. Although the use of density variables, as applied in the HEI 2005, accounts for overall energy intake, children consume much less food than adults, and their nutrient or food group density must be higher to accommodate their physiological need for higher amounts of nutrients to support healthy growth and development. Therefore, using a “one size fits all” index (early childhood to late in life) to measure the diet quality may not be prudent because it does not account for the higher nutrient need along with a lower quantity of food intake in children. To address this issue, several indexes were developed or adapted for use in child and/or adolescent populations [4]. The Youth Healthy Eating Index (YHEI) was created to target older children and adolescents and included the traditional HEI components along with additional components on breakfast consumption and family dinners [8]. The E-KINDEX was developed to rank the dietary patterns of children in relation to the risk for obesity and includes components on food intake, perceptions about food and eating behavior, and dietary practices [9]. 

Neither of those indexes addressed the specific needs of young children. The first composite diet quality index for children under five years of age was developed by Kranz and colleagues in 2006, the Children's Diet Quality Index (C-DQI) [10], which was revised to reflect the changes in dietary intake guidance in 2005 and to expand the age range from 2–5 to 2–18 years old (the Revised Children's Diet Quality Index (RC-DQI)) [11]. The RC-DQI has 13 components based on MyPyramid recommendations, American Academy of Pediatrics (AAP) fruit juice intake recommendations, the Dietary Reference Intakes (DRIs) for macronutrients and iron, and the American Dietetic Association's (ADA, now called AND) recommendations for total energy intake and television viewing. Scoring is such that children consuming within the recommended levels received full points (varying from 2.5 to 10 points, depending on the component) with reductions of points made proportionally for suboptimal intake or overconsumption (intake below or above the recommended level). Since age and gender specific recommendations were used in the development of the index, each child's age and gender were considered in point allocation as well. Examination of content validity showed that it successfully differentiated between children with varying levels of diet quality and that children with higher RC-DQI scores were significantly less likely to be at risk for chronic disease [12].

Both the HEI-2005 and RC-DQI was designed to measure total diet quality in Americans, including children 2–18 years old. A side-by-side comparison of the components and their scoring criteria for both indexes are presented in Table 1. The underlying dietary intake recommendations are very similar and the populations, to which the indexes were applied, are often dietary data from nationally representative data sets. Neither the HEI 2005 nor the RC-DQI were designed to assess diet quality in individuals and can only be used on the group level. 

To date, a comparison of possible differences between the rankings of intake levels based on the two indexes to examine has not been conducted. The indexes both include the same five dietary intake recommendations for dairy, total grains, whole grains, fruits, and vegetables. Each index also includes additional components, but they are not comparable. Thus, an examination of total HEI 2005 scores and RC-DQI scores would not yield adequate information to allow a direct comparison or examination of differences. The purpose of this paper was to examine if the scoring schemes of the five components present in both indexes affect the ranking of the dietary intake level and in which manner this difference presents itself.

This study used sociodemographic and dietary intake data of a nationally representative sample of children 2–18 years old from the National Health and Nutrition Examination Survey (NHANES) 2003–2006 to explore if children's dietary intakes were scored differentially on each of the five comparable components in the two diet quality indexes (the HEI-2005 and RC-DQI). The hypothesis tested is that although both indexes will discern between children with optimal versus suboptimal food group intake levels, the scoring of the RC-DQI, which was specifically developed to address children's dietary needs, will provide more levels of differentiation between intake levels and therefore potentially provide a higher level of detail to the researchers employing the index.

## 2. Methods

### 2.1. Data Used

Socioeconomic and dietary data from the combined survey years of 2003-2004 and 2005-2006 of the National Health and Nutrition Examination Survey (NHANES (available at http://www.cdc.gov/nchs/nhanes.htm)) were used for this study. Only children 2–18 years old for whom two days of dietary intake data are available were included (*N* = 5,936). During the survey, an adult was chosen for the household interview and reported sociodemographic information, such as gender, age, race, ethnicity, and household income.

#### 2.1.1. Sociodemographic Data

According to the interview responder's categorization, race, and ethnicity were reported as American Indian or Alaskan Native, Asian, black or African American, Native Hawaiian or Pacific Islander, white, or other non-Hispanic, Mexican American, other Hispanic. These variables were recoded to reflect the cultural eating differences in Hispanic/other (Mexican Americans, other Hispanic, other/multiethnic), nonhispanic white, and nonhispanic black children. Household income was used to differentiate households by the income-eligibility cut points for USDA food assistance programs: high income are families with household incomes ≥3.5 of the poverty income ratio (PIR), medium income defined as 1.86–3.4 PIR, and low income defined as ≤1.85 PIR. The latter group are income eligible for participation in the USDA food assistance program [13]. The PIR is truncated at 5.0; thus, values range from zero to 5.0. The PIR is used routinely to express the available income of households by expressing the total income of the household, accounting for the number of individuals living in the household.

#### 2.1.2. Dietary Intake Data

Two 24-hour dietary recalls of food consumption data are available for both 2003-2004 and 2005-2006 survey years. It is noteworthy to point out that a correction for the distribution of intakes of food groups and nutrients, such as the NCI or the Iowa State method, is not warranted in this type of analysis for a number of reasons. First, while intake distributions may affect the association between levels of consumption and the risk for chronic diseases in a manner that may lead to misclassification of individuals, thus introducing error, the comparison of two diet quality indexes scores is not affected by that threat. The analysis is based on comparing the population's intake levels of food groups twice, based on applying two distinct scoring systems to the diets of the same nationally representative sample. No individual data was generated. The aim of the study was not to rank individuals or compare individual's intake levels to disease risk factors to discern those at higher risk from those at lower risk. One might have chosen to add a calculation to correct for interperson intake variation, but no additional information would have been gained in this particular project. 

Detailed information on survey design and data collection of the NHANES data can be found elsewhere [14]. In short, children 2–6 years old reported their own diets and were assisted by a parent or caretaker, if needed. To address increasing food consumption with older age and higher level of independence due to older age and increased frequency of eating away from a caretaker (for instance while at school), data analysis was grouped for three distinct age groups: preschoolers, school-age children, and teenagers age groups, 2–5 years old (*N* = 1, 405), 6–11 years old (*N* = 1, 586), and 12–18 years old (*N* = 2, 945). Data were examined for the total sample of children as well as stratified by the three age groups.

#### 2.1.3. Diet Quality Measurement

Usual dietary intake of food groups and nutrients was estimated by calculating two-day average intakes for each child. Mixed dishes and foods were disaggregated using the MyPlate Equivalents Database (MPED) and expressed in servings (ounces or cups). The components and total scores of the HEI 2005 and RC-DQI were calculated using Stata, version 10 [15]. The original SAS Coding for the HEI was available at the Center for Nutrition Policy and Promotion (CNPP) website. The researchers converted the SAS coding into a Stata version, available at http://www.kranzlab.net/. The RC-DQI had been developed in Stata, and the programming was used to calculate scores for this study sample.

The scoring of the HEI 2005 is described in detail in the technical report [16]. The scoring of the RC-DQI is documented in a publication [11] and varies from the HEI-2005 scoring in that children's food intake was used to compare their intake to the recommended intake level by multiple authorities whereas the HEI-2005 was based on the Dietary Guidelines and the use of energy-adjusted terms.

The comparison between the two indexes and their components is shown in Table 1. Due to their nature or definition, some of the components and total scores cannot be compared directly. However, the scores for the consumption of dairy, total grains, whole grains, fruits, and vegetables were comparable and were used for this project. 

To allow comparison of HEI 2005 and RC-DQI component scores, the scoring of the RC-DQI was adjusted to reflect that of the HEI 2005, which had a maximum of 5 points for the fruits, vegetables, total grains, and whole grains components and a maximum of 10 points for the dairy component. Thus, The RC-DQI maximum scores for fruit and vegetables were reduced from 10 points to 5 points by dividing the maximum scores by two. The scores for total grains, whole grains, and dairy remained unchanged (total grains and whole grains had a maximum of 5 points and dairy a maximum of 10 points in both indexes). Although the dairy component was scored with a maximum of 10 points in both indexes, maintaining that value would have provided a larger weight of this component in the calculation of the total subscore of the five selected components; thus, in both indexes the dairy scores were divided by two, which results in a possible maximum score of 5 points. The total maximum subscore of the five HEI-2005 and RC-DQI components selected for this study was therefore 25 points; the possible minimum score was zero.

HEI 2005 and RC-DQI total subscore and component scores calculated for the population and the distribution of diet quality index points were examined in the following manner: first, the correlation of the components within each index was examined (Table 2), then the correlation between the component scores of both indexes was compared (Table 3). Lastly, the proportion of children scoring 0 (lowest possible score), between 0 and 5, and 5 (highest possible score) was described to explore the level of differentiation afforded by each index. The use of correlation coefficients between the components within each index indicates if any of the individual components predicts any or all of the other components. The comparison of the same components but between the two index, on the other hand, allows the researcher to examine the level of agreement between population's scores generated by the HEI 2005 and the RC-DQI, which was a reflection of the effect of the difference in scoring schemes, as the study population, the dietary intake levels, and the underlying dietary intake recommendations for dairy, fruits, total grain, whole grain, and vegetables are the same.

## 3. Results

The distribution of children with zero (minimum), between one and 24 points, and 25 points (maximum) was 12%, 88%, and 0% for the HEI-2005 and 0%, 100%, and 0% for the RC-DQI. 

Examination of the correlation between the five component scores under investigation within each index is shown for the total population and the three age groups in Table 2. In the HEI 2005, correlations ranged from −0.0006 to 0.3833 and included negative values. The following component pairs were significantly (*P* value <0.05) associated with each other: dairy and whole grains, dairy and fruit, whole grains and total grains, whole grains and fruit, total grains and vegetables, and total grains and fruit. There was no significant association between dairy and total grains, vegetables or whole grains and vegetables, and vegetables and fruit. Age-group specific analysis showed (Table 3) that correlation coefficients were significant for some of the components in all three age groups, but the level of association varied.

Analysis of the correlation between component scores in the RC-DQI showed that all comparisons were positive and statistically significant (*P* value 0.01) and ranged from 0.09 to 0.26. The correlations between components in the age-stratified analysis indicated that the fruit and whole grains and the fruit (0.19) and vegetables (0.22) components were correlated in the younger children.

The correlation between component scores in both indexes (Table 3) showed that associations ranged from −0.003 to 0.56, correlation coefficients were significant for milk and dairy, fruit, vegetables, total grains and whole grains. The correlations between the identical components, that is, RC-DQI dairy and HEI 2005 dairy, were positive and statistically significant at a *P* value of 0.01 (0.34 for dairy, 0.41 for whole grains, 0.17 for total grains, 0.28 for vegetables, and 0.55 for fruit).

**Figure 1:**
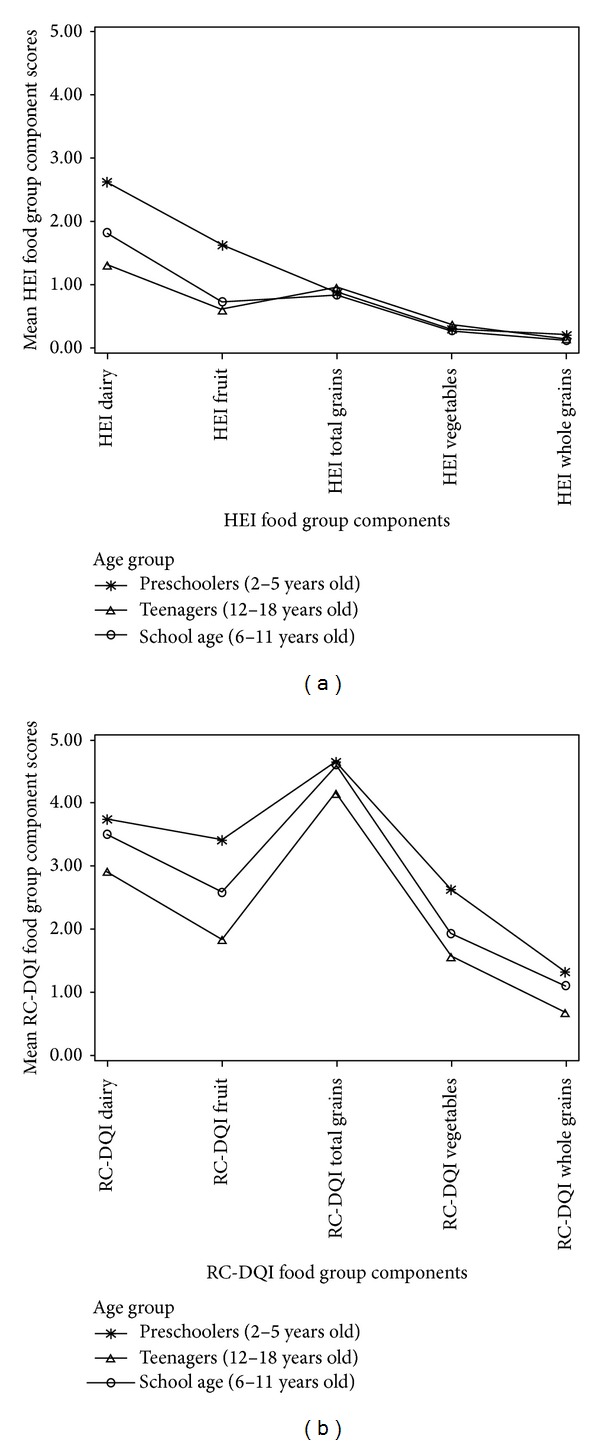
(a) Distribution of mean HEI component scores by age group. (b) Distribution of mean RC-DQI component scores by age group.

**Figure 2:**
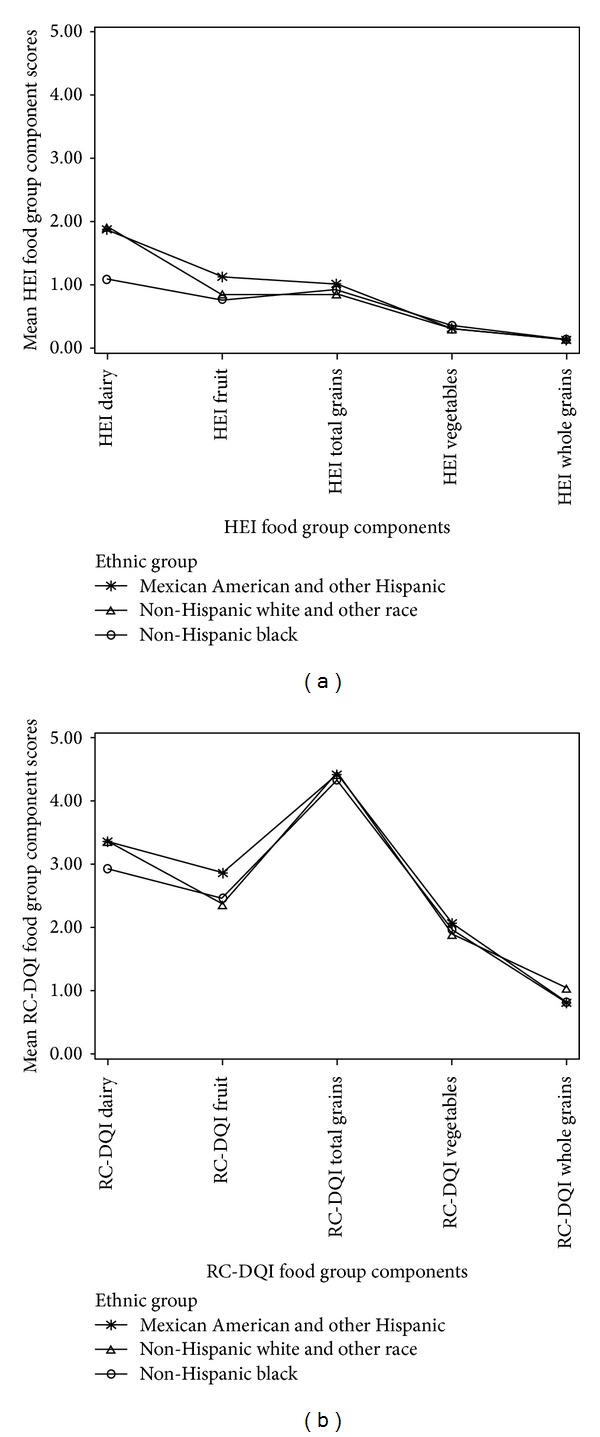
(a) Distribution of mean HEI component scores by ethnic group. (b) Distribution of mean RC-DQI component scores by ethnic group.

**Figure 3:**
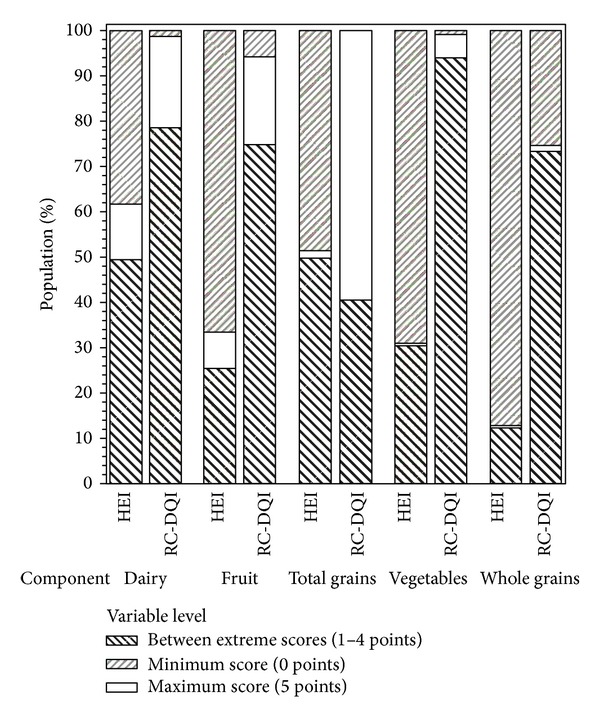
Proportion of children with the maximum, the minimum, or between the maximum and minimum diet quality index scores after applying the HEI 2005 and RC-DQI.

Analyses of the distribution of component scores by age group and ethnic group are depicted in Figures 1 and 2, which show that the RC-DQI leads to a higher differentiation for most components. This is also apparent in the comparison of the distribution of the proportion of children with the maximum score (5 points), the minimum scores (0 points), and scores between the maximum and the minimum (>0 and <5 points) (see Figure 3). With the exception of the whole grains component, the RC-DQI classifies consistently more children in the group that is between the two extremes than the HEI 2005 does.

## 4. Discussion

Measurement of DQ is complex and requires a thorough understanding of the nutritional issues affecting the population of interest [17]. While one commonly applied approach is based on capturing intake quantities and comparing them to individual food group or nutrient intake goals [18], additional information that is not captured by individual components of diets is assessed using composite assessment tools that can across the most pertinent nutritional concerns in reference to population-specific intake recommendations. This method cannot only provide information about the intake levels of food groups and nutrients, but also captures the underlying issues involved in overall diet—many of which are not known. The fact that the correlation coefficients between index components of the HEI 2005 or RC-DQI are not very high is evidence for the fact that humans do not eat in a very predictable manner, for instance, high fruit intake does not predict high milk or high vegetable intake; and using energy density controlled variables (i.e., servings of fruit per 1000 kcal of energy consumed) showed that people having higher energy intakes do not consistently eat more of everything. All this information is highly relevant to the study of dietary intake and risk for disease, such as obesity, however, the manner in which Diet Quality Index components are scored has a critical effect on the evaluation of diet quality. As this study shows, the individual components of each index were only mildly or moderately associated, indicating that each component measured independent effects. At the same time, although the population data and food group intake recommendations for children used for this study were exactly the same (two-day average intake in children ages 2–18 years old of the NHANES 2003–2006 and the MyPlate food group intake recommendations for fruits, vegetables, total grains, whole grains, and dairy), the scoring scheme of the HEI 2005 and RC-DQI resulted in different statements about diet quality in this population of American children.

Intake guidance is population sensitive and should not be applied to individuals living in other food cultures [19]; also, the HEI 2005 and the RC-DQI were developed based on population-level intake recommendations and should not be applied to the assessment of diet quality of individuals [20]. The HEI 2005 is an appropriate overall diet quality assessment tool for children [21]. However, the scoring scheme is based on complex programming steps that are not easily reproducible or conveyed to the lay person. To support the broader application of the HEI, user guidelines and coding were released for the public. The RC-DQI was also developed in the nationally representative data of 2–18 years old, but the scoring was based on nutritional issues specific to children and included intake recommendations by the American Academy of Pediatrics (in addition to the Dietary Reference Intakes and the MyPlate food group intake recommendations). Both indexes provide information on food groups and nutrients intake levels, and both have been shown to measure DQ in children.

The HEI 2005 and the RC-DQI were scored to express increased diet quality with higher component or total scores. Based on this analysis, most of the children were classified as having diets between the ideal (25 points) and very low scores (zero points) using either of the indexes; however, the HEI-2005 subscore indicated that 12% of the population had zero scores, meaning that they scored no points in either of the five components (dairy, total or whole grain, fruits, and vegetables) whereas using the RC-DQI, none of the children were scored with zero. This difference is due to the scoring scheme of the RC-DQI components, which are based on the concept of “deviation from the recommended intake.” Thus, only children who did not consume any servings of the food groups received a zero score—partial consumption resulted in proportional deduction of points. It is important to point out that children who had missing dietary data were excluded from the study analysis (they would have received a zero score).

The examination of the associations between component scores within the HEI 2005 showed that several of the components were not related to each other and, although not significant, an increase in one component could be associated with a decrease of another; that is, dairy, whole grains, or fruit scores were associated with lower vegetable scores. The association between the RC-DQI components, on the other hand, showed that increased component scores were consistent in all five components and had at least moderate and statistically significant correlation coefficients for all comparisons.

Interestingly, the correlation coefficients between the two indexes showed a wide range of associations, including positive, significant, negative, and nonsignificant associations. For instance, while the association between the component scores of whole grains and fruit was at least moderate, the association between total grains or vegetable scores were much lower. Thus, although the data analysis was based on the same sample of a nationally representative group of children 2–18 years old and their estimated two-day average dietary intake of food groups, the scoring scheme of the two indexes resulted in differential evaluation of the diet quality.

Results from this study demonstrate the importance of understanding the relationship between dietary intake of food groups and nutrients. Based on the scoring method employed, the calculated dietary quality index scores varied greatly in the evaluation of the population's diet quality. The RC-DQI scores led to higher proportions of the population with scores between the two extremes of maximum and minimum scores for the components or the subscore. Thus, depending on the research question, researchers interested in estimating overall quality of children ages 2–18 must consider the desired level of differentiation of diet and select the appropriate index. Furthermore, depending on the aims of the study, it might be more appropriate to employ only individual component scores rather than the total score of a diet quality index. 

In recent years, the diet quality of the American population has become a public health issue of great concern [22]. However, measuring the concept of diet quality is complex, and more research is needed to identify some of the underlying factors leading to the dietary intake behaviors of children. Public health measures, such as taxing fast food [23–25] or using simple nutrition guidance, such as the “traffic-light” labels [26], are considered by some as effective tools to improve children's diet quality; however, it remains to be seen if the implementation of the policies translates into changes in dietary intake behavior. Nonetheless, the measurement of diet quality will remain a critical issue in nutrition monitoring, and more and detailed studies need to be conducted to help advance the science.

## Figures and Tables

**Table:** (a)

HEI 2005*			
Component	Max points	Max score (per 1000 kcal)	Min score
			
Total fruit	5	≥0.8 c equiv.	No fruit
Whole fruit (no juice)	5	≥0.4 c equiv.	No whole fruit
Total vegetables	5	≥1.1 c equiv.	No vegetables
Dark green and orange vegetables and legumes	5	≥0.4 c equiv.	No dark green or orange vegetables or legumes
Total grains	5	≥3.0 oz equiv.	No grains
Whole grains	5	≥1.5 oz equiv.	No whole grains
Milk	10	≥1.3 c equiv.	No milk
Meat and beans	10	≥2.5 oz equiv.	No meat or beans
Oils	10	≥12 grams	No oil
Saturated fat	10	≤7% of energy	≥15% of energy
Sodium	10	≤0.7 gram	≥2.0 g per 1000 kcal
SoFAAS**	20	≤20% of energy	≥50% of energy

*Excerpted from: P. M. Guenther, S. M. Krebs-Smith, J. Reedy, P. Britten, W. Y. Juan, M. Lino, A. Carlson, H. A. Hiza, and P. Basiotis: Healthy Eating Index-2005 fact sheet. Alexandria, VA: United States Department of Agriculture, Center for Nutrition Policy and Promotion; 2006.

**Calories from solid fats, alcoholic beverages, and added sugars.

**Table:** (b)

Component	Score	Age (years)																	Scoring criteria
		2	3	4	5	6	7	8	9	10	11	12	13	14	15	16	17	18	
Added sugar^a^	10	≤10% total energy																	WHO
Fat^a^	2.5	30–40%		25–35% total energy															AMDR
Linoleic acid (18_2)^a^	2.5	≤5–10% of total energy																	(*ω*-6 f.a.)
Linolenic acid (18_3)^a^	2.5	0.6–1.2% of total energy																	(*ω*-3 f.a.)
DHA and EPA^a^	2.5	≤10% of *α*-linolenic acid																	(More potent *ω*-3 f.a.)
Total grains^a,c^	5	3	3	4	4	4	4	4	6	6	6	6	6	7	7	7	7	7	Food groups
Whole grains^a,c^	5	1.5	1.5	2	2	2	2	2	3	3	3	3	3	3.5	3.5	3.5	3.5	3.5	Food groups
Fruit^a,c^	10	1.5	1.5	1.5	1.5	1.5	1.5	1.5	2	2	2	2	2	2	2	2	2	2	Food groups
Vegetable^a,c^	10	1	1	2	2	2	2	2	3	3	3	3	3	4	4	4	4	4	Food groups
Excess juice^a^	10	6	6	6	6	6	12	12	12	12	12	12	12	12	12	12	12	12	AAP age-appropriate limit
Dairy^a,c^	10	2	2	2	2	2	2	2	3	3	3	3	3	3	3	3	3	3	Food groups and AAP
Iron^b^		≤3.0		≤4.1					≤5.7					≤7.9					≤EAR = 0 points
	10	3.1–6.9		4.2–9.9					5.8–7.9					8.0–14.9					EAR-RDA = 5 points
		≥7		≥10					≥8					≥15					≥RDA = 10 points
TV/energy ^a,d^	10	EER: 1072	EER: 1080	EER: 1133	EER: 1189	EER: 1247	EER: 1298	EER: 1360	EER: 1415	EER: 1470	EER: 1538	EER: 1617	EER: 1684	EER: 1718	EER: 1731	EER: 1729	EER: 1710	EER: 1690	2 hours TV Energy ± 10% of EER

Total Points	**90**																		

^a^Continuous score.

^b^Categorical variable based on less than EAR (0 pts), EAR-RDA (5 pts), or meets RDA (10 pts).

^c^Dietary Guidelines MyPyramid food group patterns by age-appropriate energy patterns (page 292).

^d^Combined score: (TV score + energy score)/2.

TV score: ≤2 hours of TV = 10 points.

EER score = 0.9 ∗ EER to 1.1 ∗ EER = 10 points, otherwise for overconsumption 10 points − ((actual intake/highest EER point ∗ 100))%, for underconsumption 10 − (actual intake/lowest EER point ∗ 100)%.

**Table:** (c)

Component	Score	Age (years)																	Scoring criteria
		2	3	4	5	6	7	8	9	10	11	12	13	14	15	16	17	18	
Added sugar^a^	10	≤10% of total energy intake																	WHO
Fat^a^	2.5	30–40%		25–35%															AMDR
Linoleic acid (18_2)^a^	2.5	≤5–10% of total energy																	(*ω*-6 f.a.)
Linolenic acid (18_3)^a^	2.5	0.6–1.2% of total energy																	(*ω*-3 f.a.)
DHA and EPA^a^	2.5	≤10% of *α*-linolenic acid																	(More potent *ω*-3 f.a.)
Total grains^a,c^	5	3	3	5	5	5	5	5	7	7	7	7	7	9	9	9	9	9	Food groups
Whole grains^a,c^	5	1.5	1.5	2.5	2.5	2.5	2.5	2.5	3.5	3.5	3.5	3.5	3.5	4.5	4.5	4.5	4.5	4.5	Food groups
Fruit^a,c^	10	1.5	1.5	2	2	2	2	2	2	2	2	2	2	3	3	3	3	3	Food groups
Vegetable^a,c^	10	1	1	2	2	2	2	2	4	4	4	4	4	4	4	4	4	4	Food groups
Excess juice^a^	10	6	6	6	6	6	12	12	12	12	12	12	12	12	12	12	12	12	AAP age-appropriate limit
Dairy^a,c^	10	2	2	2	2	2	2	2	3	3	3	3	3	3	3	3	3	3	Food groups and AAP
Iron^b^		≤3.0		≤4.1					≤5.9					≤7.7					≤EAR = 0 points
	10	3.1–6.9		4.2–9.9					6.0–7.9					7.8–10.9					EAR-RDA = 5 points
		≥7		≥10					≥8					≥11					≥RDA = 10 points
TV/energy^a,d^	10	EER: 1120	EER: 1162	EER: 1215	EER: 1275	EER: 1328	EER: 1393	EER: 1453	EER: 1530	EER: 1601	EER: 1691	EER: 1798	EER: 1935	EER: 2090	EER: 2223	EER: 2320	EER: 2366	EER: 2383	2 hours TV Energy ± 10% of EER

Total Points	**90**																		

^a^Continuous score.

^b^Categorical variable based on less than EAR (0 pts), EAR-RDA (5 pts), or meets RDA (10 pts).

^c^Dietary Guidelines MyPyramid food group patterns by age-appropriate energy patterns (page 292).

^d^Combined score: (TV score + energy score)/2.

TV score: ≤2 hours of TV = 10 points.

EER score = 0.9 ∗ EER to 1.1 ∗ EER = 10 points, otherwise for overconsumption 10 points − ((actual intake/highest EER point ∗ 100))%, for underconsumption 10 − (actual intake/lowest EER point ∗ 100)%.

Imputation data, if TV/screen time was missing in the dataset:

2–4 year olds: 2 hours (28).

2–6 year olds: 2 hours/d.

7–9 year olds: 3.5 hours/d (includes video and videogames) (29).

8–10 year olds: 4.5 hours/d.

11–14 year olds: 4.5 hours/d.

15–18 year olds: 3.5 hours/d (30).

10–19 year olds: 3 hours/d (31).

6–19 year olds: 4–6 hours/d (32).

Use for RC-DQI coding:

2–6 year olds: 2 hours/d.

7–14 year olds: 4 hours/d.

15–18 year olds: 3.5 hours/d.

**Table:** (a) HEI 2005

Components	Fruits	Vegetables	Total grain	Whole grain
Total sample (*N* = 5,936)				
Milk/dairy	0.0891**	−0.0288	0.0317	0.0545*
Fruit		−0.0359	0.0711**	0.0726**
Vegetables			0.1017**	−0.0006
Total grains				0.3007**
Whole grains				

2–5 year olds (*N* = 1,405)				
Milk/dairy	0.0027	−0.0085	−0.0176	−0.0254
Fruit		−0.0596	0.1079	0.1007**
Vegetables			0.0603	−0.0259
Total grains				0.3833**
Whole grains				

6–11 year olds (*N* = 1,586)				
Milk/dairy	0.0198	−0.0676*	−0.0164	−0.0033
Fruit		0.0183	0.1185*	0.0722
Vegetables			0.1267*	0.0207
Total grains				0.3027**
Whole grains				

12–18 year olds (*N* = 2,945)				
Milk/dairy	0.0531	0.0099	0.1157**	0.1347**
Fruit		−0.0481	0.0311	0.0192
Vegetables			0.0981**	0.0015
Total grains				0.2661**
Whole grains				

**Table:** (b) RC-DQI in the total sample and three mutually exclusive age groups

Components	Fruits	Vegetables	Total grain	Whole grain
Total sample (*N* = 5,936)				
Milk/dairy	0.1326**	0.0938**	0.2522**	0.1585**
Fruit		0.2025**	0.1513**	0.2274**
Vegetables			0.2056**	0.1070**
Total grains				0.2560**
Whole grains				

2–5 year olds (*N* = 1,405)				
Milk/dairy	0.0312	−0.0047	−0.0024	0.0060
Fruit		0.2165**	0.0779	0.1881**
Vegetables			0.1496**	0.0454
Total grains				0.2203**
Whole grains				

6–11 year olds (*N* = 1,586)				
Milk/dairy	0.0820	0.0488	0.2442**	0.1980**
Fruit		0.0632	0.0760*	0.1738**
Vegetables			0.1305**	0.0491
Total grains				0.2510**
Whole grains				

12–18 year olds (*N* = 2,945)				
Milk/dairy	0.0533	0.0362	0.2619**	0.1092**
Fruit		0.0676	0.0926*	0.1469**
Vegetables			0.1950**	0.0427
Total grains				0.2067**
Whole grains				

*significant at *P* value < 0.05.

**significant at *P* value < 0.01.

**Table 3:** Correlation coefficients (*r*-square) between the comparable component scores of the HEI 2005 and RC-DQI in the total sample and three mutually exclusive age groups.

HEI 2005					
Components	RC-DQI				
	Milk/Dairy	Fruits	Vegetables	Total grain	Whole grain
Total sample (*N* = 5,936)					
Milk/dairy	0.3381**	0.1217**	0.0350	0.0337	0.1569**
Fruit	0.0297	0.5506**	0.0838**	−0.0058	0.1418**
Vegetables	−0.0724**	−0.0344	0.2769**	−0.0843**	−0.0275
Total grains	−0.0469*	0.0024	−0.1004**	0.1659**	0.0376
Whole grains	0.0066	0.0536**	−0.0351	0.0453**	0.4145**

2–5 year olds (*N* = 1,405)					
Milk/dairy	0.2036**	0.0045	−0.0467	−0.0772	0.0432
Fruit	−0.0252	0.5555**	0.0481	−0.0007	0.1871**
Vegetables	−0.0709	−0.0520	0.2601**	−0.0511	−0.0417
Total grains	−0.0668	0.0186	−0.0953*	0.1867**	0.1871**
Whole grains	−0.0682	0.0462	−0.1035*	0.0686**	0.4263**

6–11 year olds (*N* = 1,586)					
Milk/dairy	0.2769**	0.0535	−0.0718	−0.0263	0.0888
Fruit	−0.0247	0.4800**	−0.0334	−0.0433	0.1057*
Vegetables	−0.0492	0.0129	0.2948**	−0.0737	−0.0239
Total grains	−0.0691	0.0636	−0.0897**	0.1573**	−0.0098
Whole grains	−0.0121	0.0918*	0.0130	0.0613**	0.3613**

12–18 year olds (*N* = 2,945)					
Milk/dairy	0.3702**	0.0251	−0.0483	0.0003	0.1813**
Fruit	−0.0062	0.5476**	0.0039	−0.0835*	0.0029
Vegetables	−0.0659	−0.0292	0.3633**	−0.0797*	0.0053
Total grains	−0.0071	−0.0246	−0.1118**	0.1942**	0.0106
Whole grains	0.0504	0.0115	−0.0525	0.0246	0.4781**

*significant at *P* value < 0.05.

**significant at *P* value < 0.01.
